# HIV-associated extranodal NK/T cell lymphoma, nasal type: a case report and literature review

**DOI:** 10.3389/fimmu.2026.1954471

**Published:** 2026-10-07

**Authors:** Junjie Wang, Lingyao Du, Tian Dong, Sha Zhao, Jie Ji, Hong Tang

**Affiliations:** 1 Center of Infectious Diseases, West China Hospital of Sichuan University, Chengdu, China; 2 Department of Hematology, West China Hospital of Sichuan University, Chengdu, China; 3 Department of Histology, West China Hospital of Sichuan University, Chengdu, China

**Keywords:** acquired immune deficiency syndrome, case report, extranodal NK/T-cell lymphoma, MDT, treatment

## Abstract

Extranodal NK/T-cell lymphoma (ENKTCL) is a rare aggressive lymphoma whose management is complicated by HIV-1 co-infection due to drug-drug interactions and overlapping toxicities, specifically the convergent hepatorenal and hematological toxicities resulting from the concurrent use of chemotherapeutic regimens and ART. We report a middle-aged HIV-1 positive man (CD4^+^ T cells:198/μL, HIV-1 RNA:9.70×10³ copies/mL) with stage IE ENKTCL nasal type, whose initial nasal congestion was repeatedly misdiagnosed as sinusitis. An integrase strand transfer inhibitor-based (INSTIs) ART was unaffordable, and efavirenz was contraindicated by prior allergy, necessitating continuation of lopinavir/ritonavir-based ART despite its CYP3A4 inhibition. A multidisciplinary team guided concurrent therapy with two full-dose cycles of P-GemOxD (gemcitabine, pegaspargase, oxaliplatin, dexamethasone) followed by consolidative radiotherapy (20 fractions). The patient tolerated treatment without grade ≥ 3 adverse events. Post-therapy PET-CT confirmed complete metabolic response, with HIV-1 RNA undetectable. At 9-month follow-up, remission persisted and CD4^+^ T cells rose to 255/μL. This case illustrates that an MDT model offers a practical management framework for delivering full-dose P-GemOxD chemotherapy with non-INSTIs when INSTI-based regimens are not accessible. Our experience provides a reference strategy for managing the therapeutic complexities of concurrent HIV and malignancy.

## 1 Introduction

Human immunodeficiency virus type I (HIV-1) infection remains a global health burden. While highly active antiretroviral therapy (ART) has dramatically reduced opportunistic infections, HIV-associated cancers have emerged as a leading cause of mortality in this population. In the past, Kaposi sarcoma, aggressive non-Hodgkin lymphoma, and invasive cervical cancer were considered “AIDS-defining cancers”. However, with the evolution of HIV management, the distinction between AIDS-defining cancer (ADC) and non-AIDS-defining cancers (NADCs) has become less central, and the broader term “HIV-associated cancers” is increasingly used (1). Among the spectrum of NADCs, which collectively account for a growing proportion of HIV-associated cancers, extranodal NK/T-cell lymphoma (ENKTCL) represents a rare but clinically challenging entity. Given its aggressive clinical course, strong association with Epstein-Barr virus (EBV), and unique therapeutic challenges, this malignancy warrants specific attention in the HIV-infected population.

ENKTCL is a rare and aggressive subtype of non-Hodgkin lymphoma, characterized by distinct geographic and ethnic predilection, with higher prevalence in East Asia and South America. The nasal type (ENKTCL-NT), accounting for approximately 80% of cases, typically involves the upper aerodigestive tract and is strongly associated with Epstein–Barr virus infection, exhibiting histopathological features of vascular destruction and coagulative necrosis. Despite current therapeutic strategies, the 5-year overall survival rate for ENKTCL-NT remains suboptimal, ranging from 40% to 50%, heavily dependent on the initial disease stage (2, 3).

The co-occurrence of HIV-1 and ENKTCL presents unique therapeutic challenges. The immunocompromised state of PLWH, compounded by potential pharmacokinetic interactions between ART and chemotherapeutic regimens, complicates treatment selection and dosing. In this context, a multidisciplinary team (MDT) approach has been increasingly advocated to optimize individualized management, particularly for rare malignancies with complex comorbidities. However, clinical experience regarding the application of MDT-guided chemotherapy in HIV-associated ENKTCL remains scarce, and optimal treatment strategies have yet to be established. Herein, we report a case of HIV-1-positive patient with ENKTCL to contribute to the limited body of evidence on this rare entity.

## 2 Case description

A middle-aged HIV-1 positive man presented with a 7-month history of progressive nasal congestion and intermittent fever. His vital signs on admission were stable: temperature, 37.2 °C; pulse, 85 bpm; respiration, 19 bpm; and blood pressure, 109/74 mmHg. No enlarged superficial lymph nodes were palpable, although tenderness was noted in the sinus area, with no other abnormalities. HIV-1 had been diagnosed six months earlier (HIV-1 RNA: 9.70×10³ copies/mL; CD4^+^T cells: 198 cells/μL), but ART with TDF + 3TC+EFV was halted within one month due to severe rash, requiring a switch to AZT + 3TC+LPV/r. The patient had been receiving SMZ-TMP (SMZ 400mg +TMP 80mg, one tablet per day) as Pneumocystis prophylaxis since the initial HIV-1 diagnosis. Despite this, nasal symptoms worsened without response to prolonged antibiotics, prompting admission for biopsy (**Figure 1**, **Figure 2**), which unexpectedly revealed ENKTCL-NT. Bone marrow biopsy revealed focally reduced hematopoietic cellularity with scattered intermediate-sized atypical cells exhibiting irregular nuclear contours (**Figure 2**). However, concurrent bone marrow flow cytometry demonstrated no detectable immunophenotypic abnormalities, and whole-body PET-CT showed no increased fluorodeoxyglucose uptake in the bone marrow (**Figure 3**). Given the concordant negative findings from both flow cytometry and PET-CT, the morphologic atypical cells were interpreted as reactive changes. The marrow was therefore deemed uninvolved, and the disease was staged as IE according to the Lugano classification. This lymphoma subtype is recognized as a rare aggressive malignancy with a 5-year survival of only 40–50%, even in immunocompetent hosts. The patient exhibited mild leukopenia and erythrocytopenia (thrombocytes were normal), elevated β2-microglobulin, concurrent HIV/EBV infection, and a CD4 count of 198 cells/μL, meeting the immunodeficient state (**Table 1**).

**Figure 1:**
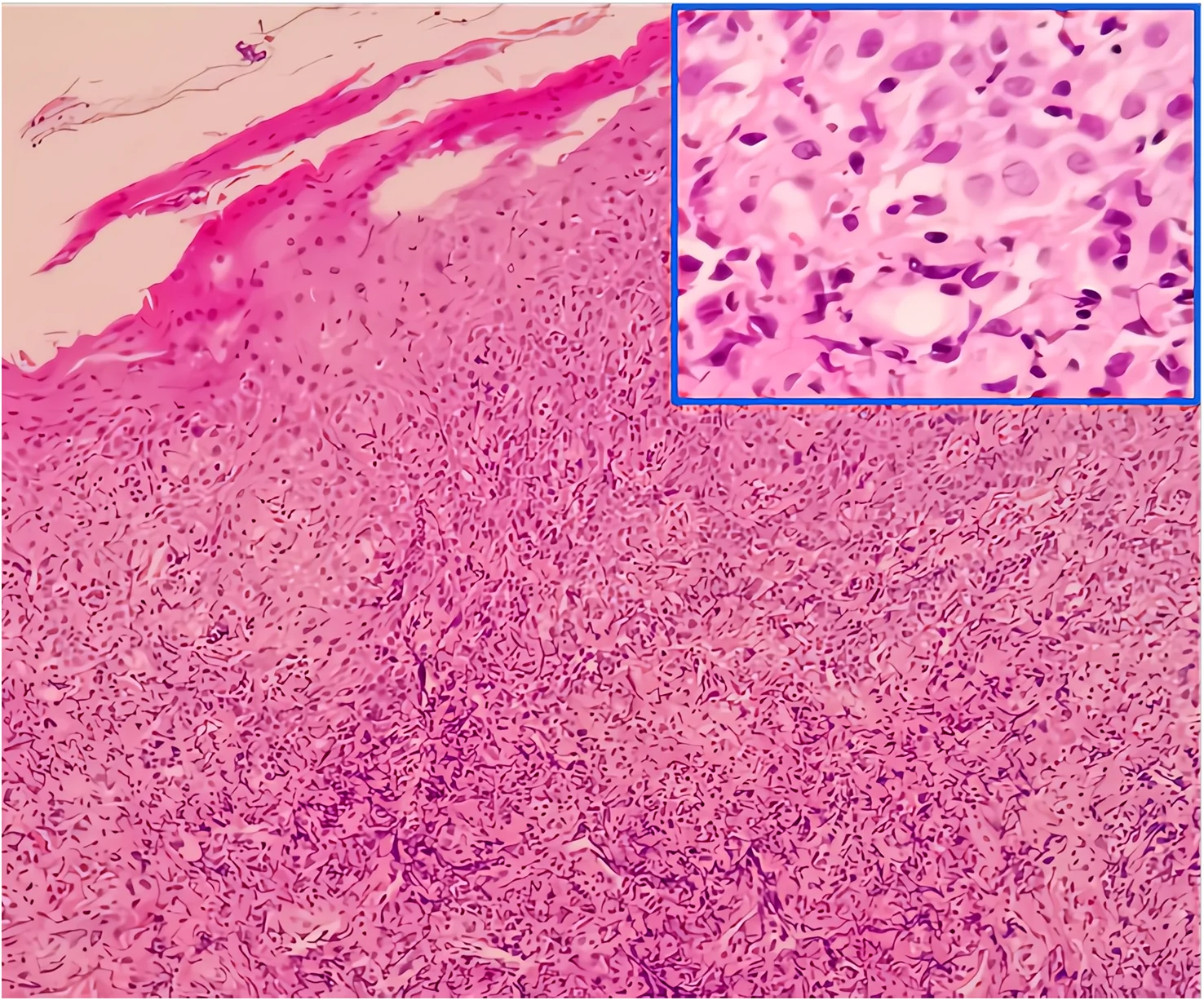
HE stained tissue from nasal endoscopy biopsy. Revealed diffuse infiltration of moderately large, morphologically uniform lymphoid cells accompanied by necrosis and inflammatory exudate.

**Figure 2:**
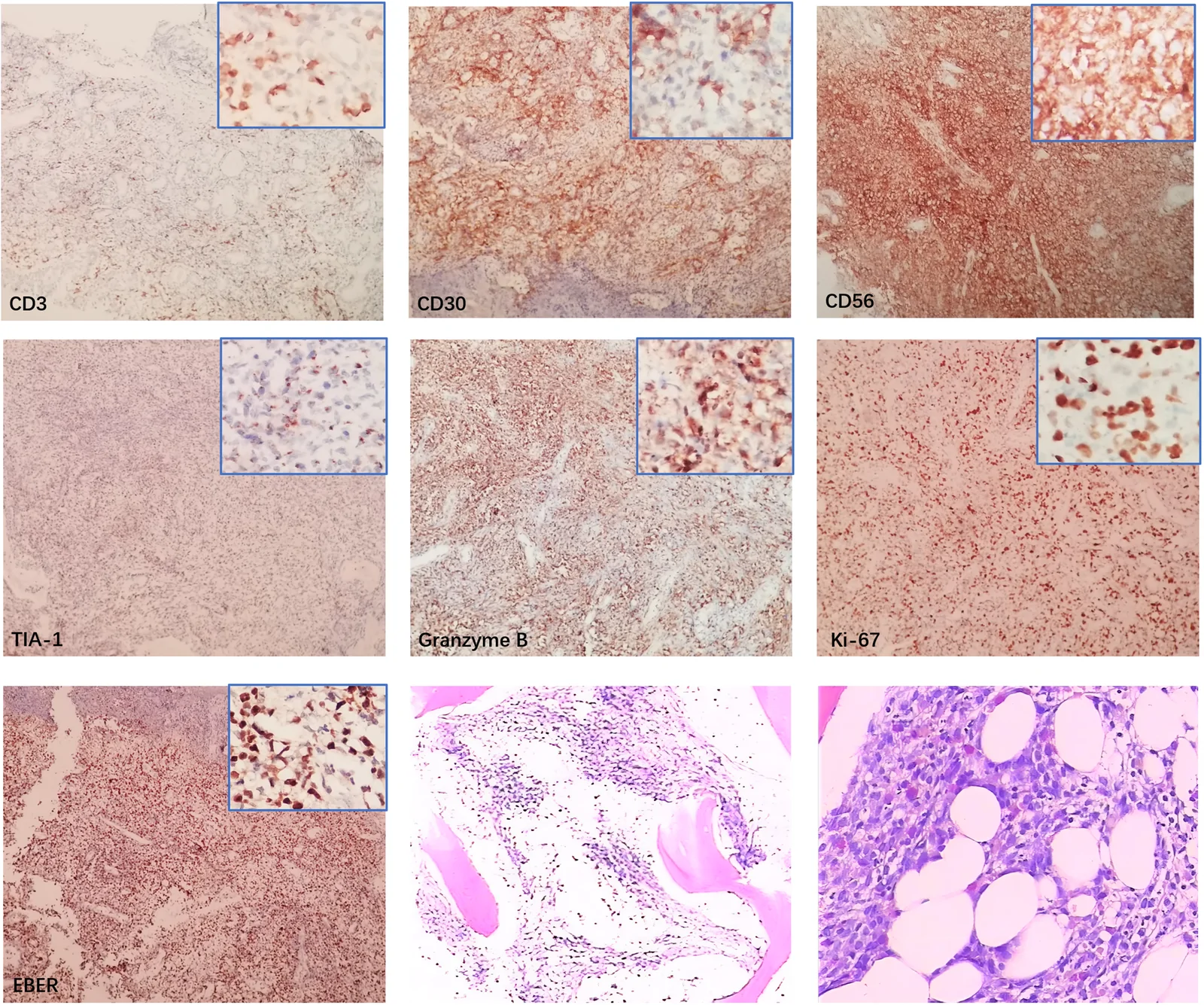
Immunohistochemical and *in situ* hybridization profiles of the nasal lesion and bone marrow histology. No. 1–7 Representative immunohistochemical and EBER *in situ* hybridization (ISH) staining of the nasal endoscopic biopsy. CD3(+), CD30(+), CD56(+), TIA-1(+), Granzyme B (+), Ki-67/MIB-1(+, approximately 90%), and EBER1/2-ISH (+). No.1 CD3 cytoplasmic antibody shows partial positivity (polyclonal antibody). No.2 CD30 shows variable (weak to strong) positive staining in the majority of cells. No.3 CD56 shows diffuse strong positivity. No.4 TIA-1 shows diffuse cytoplasmic dot-like positivity. No.5 Granzyme B shows diffuse positivity. No.6 Ki-67 shows a high proliferation index, accounting for approximately 90%. No.7 EBER1/2-ISH showed diffuse and strong positivity in virtually all tumor cells, supporting an EBV-driven etiology. The neoplastic cells were entirely negative for CD20, CD5, CD4, CD8, and CD38 (data not shown). No.8 Bone marrow smear reveals reduced hematopoietic cellularity. On low-power microscopy, atypical cells are arranged in focal sheets between the trabeculae. No.9 Within the adipose tissue, intermediate-sized atypical cells are identified, exhibiting irregular nuclear contours, inconspicuous nucleoli, and scattered nuclear debris. (8–9 images tissue from the bone marrow).

**Figure 3:**
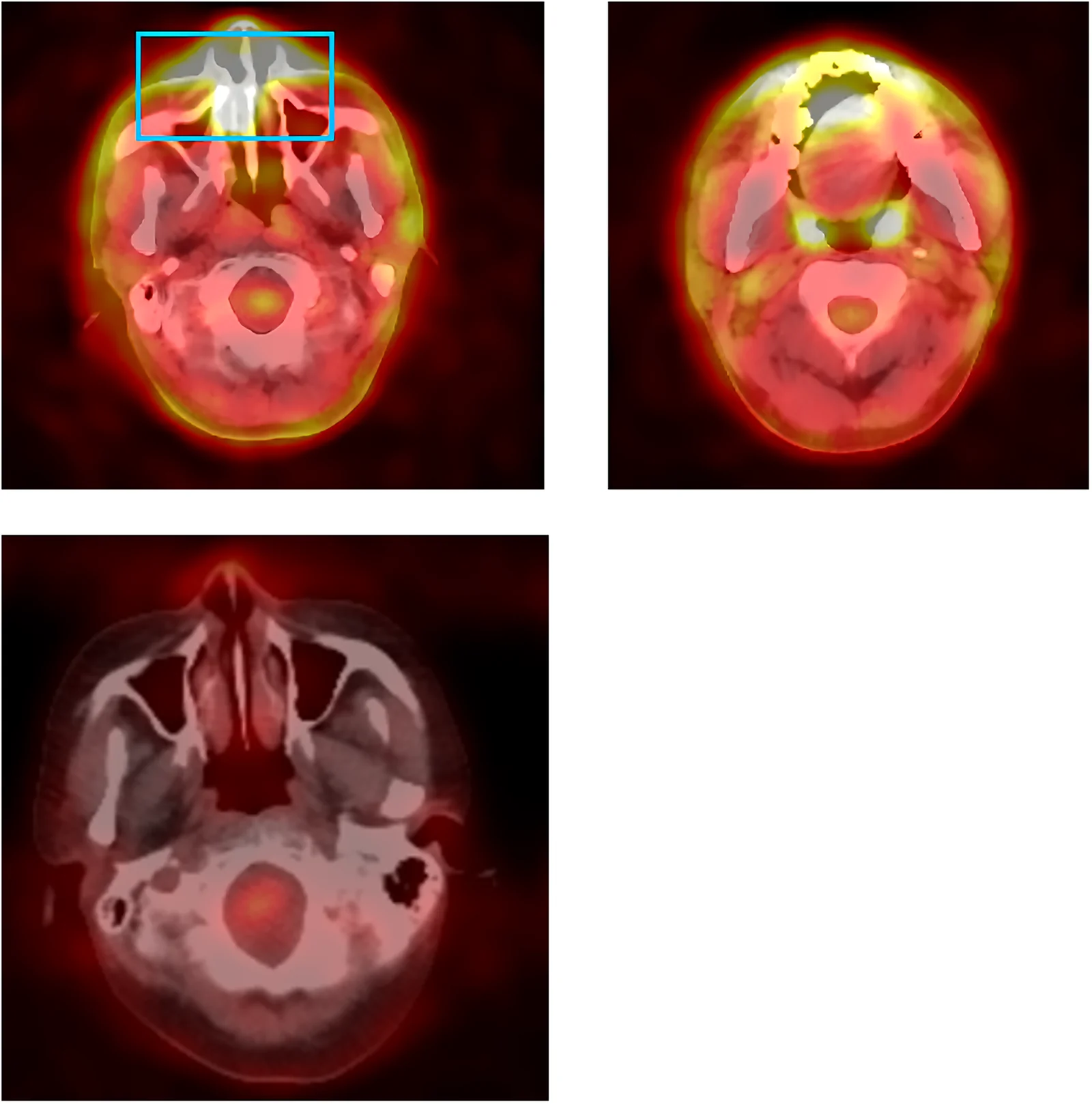
PET-CT. Top panel (before treatment): A soft tissue shadow in the right nasal cavity with abnormal increased glucose metabolism was detected, and lymphoma invasion was considered (blue square). The bilateral cervical lymph nodes and Waldeyer’s ring showed increased glucose metabolism and multiple inflammatory or reactive changes. Right maxillary sinusitis. No definite abnormal hypermetabolism was observed in the remainder of the body, including the bone marrow, liver, spleen, and mediastinum. Bottom panel (after treatment): The glucose metabolic activity of the local lesion was significantly inhibited, the tumor activity was remarkably reduced, and no obvious residual abnormal hypermetabolism was observed. No new hypermetabolic lesions were detected elsewhere.

**Table 1:** Auxiliary examination-admission.

Auxiliary examination	Result
Red blood cell count(RBC)	3.72*10^12/L
Platelet count(PLT)	270*10^9/L
White blood cell count(WBC)	3.18*10^9/L
Lymphocyte percentage	17.3%
Percentage of monocytes	11.3%
Estimated glomerular filtration rate(eGFR)	124.68 mL/min/1.73 m^2^
TP-Ab	–
HCV-Ab	–
HBsAb	+
PT	9.8 s
APTT	23.0 s
Serum β2 microglobulin	3.1100 mg/L
EB-DNA	7.62*10^2 copies/mL
HIV-1 RNA	1.34*10^2 copies/mL
TORCH-IgG	–
TORCH-IgM	–
CMV-DNA	–
Absolute CD4 cell count	198 cell/uL
Absolute CD8 cell count	579 cell/uL
CD4/CD8 ratio	0.34

−, negative; +, positive.

The case posed three intersecting challenges. Firstly, according to Chinese guidelines, the optimal ART for concurrent cancer therapy is INSTI-based regimens, but the patient could not afford it; EFV was contraindicated due to prior allergy, leaving LPV/r as the only viable option — yet LPV/r is a strong CYP3A4 inhibitor, raising concerns about altered chemotherapy metabolism. Secondly, the baseline CD4^+^T cell counts of 198 cells/μL placed the patient in a low-immunity stratum, raising substantial concern that full-dose cytotoxic chemotherapy could precipitate severe and protracted myelosuppression, thereby increasing susceptibility to life-threatening infections. However, any empiric dose reduction of the P-GemOxD regimen might have undermined the dose-intensity necessary for durable remission in this aggressive lymphoma. Thirdly, ENKTCL-NT requires L-asparaginase-based regimens, which carry hepatotoxicity and coagulopathy risks that could be amplified by concurrent ART.

After MDT deliberation, we chose the P-GemOxD regimen (gemcitabine, pegaspargase, oxaliplatin, dexamethasone) — balancing efficacy against ENKTCL, tolerability in an immunosuppressed host, and affordability. We administered full doses despite the CD4^+^T cells nadir, with intensified monitoring for hepatotoxicity and myelosuppression. Two cycles were given at 2-week intervals, followed by consolidative radiotherapy (20 fractions). (pegaspargase 2500U/m², day 1; gemcitabine 800mg/m², day 1; oxaliplatin 100mg/m², day 1; dexamethasone 20mg day1-5) (**Figure 4**). Remarkably, the patient tolerated treatment without grade ≥ 3 adverse events (**Supplementary Figure S1**). At completion, imaging confirmed complete metabolic response. At 9 months post-radiotherapy, CD4^+^T cells had recovered to 255 cells/μL, with sustained virologic suppression, EBV-DNA remained undetectable and maintained remission.

**Figure 4:**
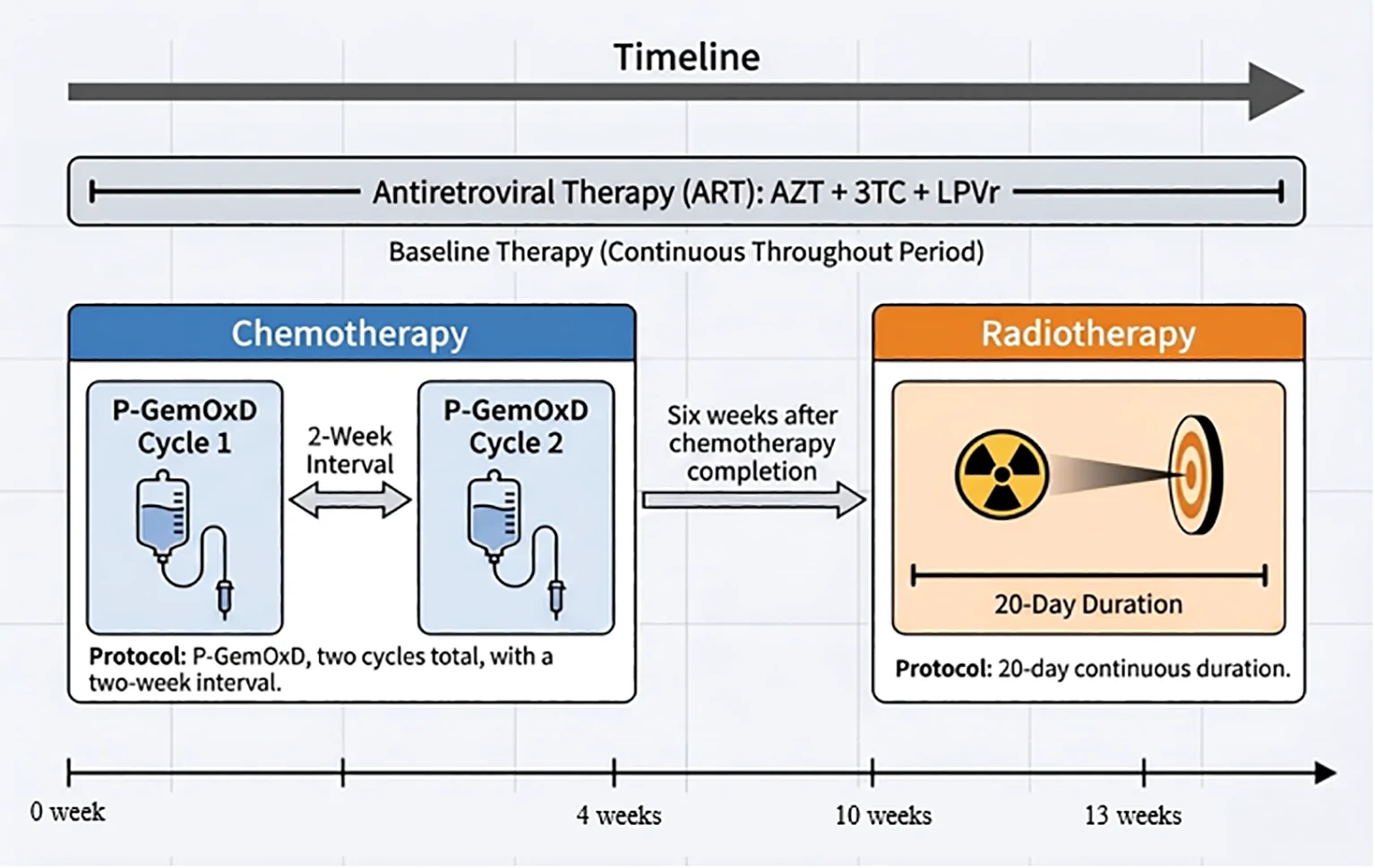
Timeline of the patient’s treatment. The patient received continuous ART comprising AZT, 3TC, and LPV/r as the baseline background regimen throughout the treatment period. Concurrent antitumor treatment consisted of two cycles of the P-GemOxD chemotherapy regimen, administered at a 2-week interval. Radiotherapy with a continuous 20-day duration was subsequently initiated at a 6-week interval following the completion of chemotherapy.

## 3 Patient perspective

The patient initially attributed his nasal symptoms to chronic sinusitis. The HIV-1 diagnosis and subsequent rash from first-line ART in national free program caused significant distress. He declined INSTI-based ART due to financial reasons and chose a protease inhibitor-based regimen instead. Upon ENKTCL diagnosis, he was distressed but agreed to chemotherapy after multidisciplinary counseling.

## 4 Discussion

HIV-1 associated lymphomas have emerged as the primary cause of cancer-related mortality in PLWH on effective ART, yet ENKTCL-NT remains uncommon in this population (4, 5). This rarity, coupled with the unique management challenges posed by concurrent HIV-1 infection, makes each reported case valuable for accumulating evidence. Our study enhances the limited research by demonstrating that full-dose P-GemOxD chemotherapy can be safely administered with LPV/r-based ART in a patient whose CD4^+^T cells count is around 200/μL—a scenario where clinical guidelines offer little concrete guidance (4).

A systematic review of the literature identified 93 cases of HIV-associated NK/T-cell lymphomas, with a median age of 38 years, a 4:1 male predominance, and a median CD4^+^T cells count of 184 cells/μL at presentation; 53% presented with stage IV disease, and median overall survival was only 1.1 years, though HAART was rarely used in that series (6). In the modern ART era, a case report by Fan et al. documented durable remission (>103 months) in an AIDS-associated ENKTCL patient treated with L-asparaginase-based chemotherapy and consolidative radiotherapy (4). Regarding treatment selection, CHOP is ineffective in ENKTCL due to P-glycoprotein-mediated multidrug resistance, whereas L-asparaginase-based regimens which include SMILE, AspaMetDex, and P-GemOx are now preferred (7, 8).

ENKTCL-NT is closely linked to EBV infection, and PLWH are especially vulnerable to EBV-induced lymphoproliferation because of weakened T-cell monitoring. The rate of misdiagnosing ENKTCL-NT is concerningly high, reaching up to 71.7% among 120 patients, with initial symptoms often being mistaken for chronic sinusitis or rhinitis (9). Our patient’s seven-month diagnostic journey, with multiple failed treatments before biopsy, echoes this pitfall. Given the high misdiagnosis rate of ENKTCL-NT and the low risk of endoscopic biopsy, we suggest that early tissue diagnosis should be prioritized over prolonged empirical therapy in HIV-1 positive patients with unexplained, persistent nasal symptoms.

In HIV-1 positive patients, lymphoma symptoms often overlap with opportunistic infections, and atypical imaging findings further complicate diagnosis. Moreover, healthcare stigma and inequity may delay access to standard services, and initial treating physicians—even in specialty departments—may opt for conservative therapy out of concern for infection, potentially compromising curability (10). The differential for this patient’s refractory nasal symptoms included sinusitis, EBV-driven lymphoproliferative disease, and other HIV-related malignancies such as Kaposi’s sarcoma. However, the failure of empirical antibiotic therapy, couple with HIV-1 infection and elevated EBV-DNA titer, shifted the leading diagnosis toward ENKTCL-NT, which was subsequently verified by nasal biopsy and immunohistochemical staining. To address these barriers, our institution established a dedicated MDT for HIV-associated malignancies, integrating expertise from hematology, infectious diseases, radiology, pathology, and otolaryngology. This collaborative framework enhances diagnostic efficiency, improves disease awareness across specialties, and ensures that treatment decisions are not disproportionately influenced by any single concern—whether infection risk, drug interaction, or affordability.

Building upon this literature background, our MDT next systematically addressed two interconnected therapeutic decisions: ART selection and chemotherapy regimen choice. For our patient, the MDT first addressed ART selection. Chinese guidelines recommend INSTI-based regimens for PLWH undergoing cancer therapy to avoid pharmacokinetic interactions, but INSTIs were unaffordable for this patient (11). EFV was contraindicated due to prior severe hypersensitivity, leaving LPV/r, a strong CYP3A4 inhibitor, as the only viable option. Despite theoretical concerns about altered dexamethasone metabolism, we observed no unexpected toxicities or reduced efficacy, suggesting that LPV/r-based ART remains a safe alternative under close monitoring when INSTIs are unavailable.

Regarding chemotherapy, the optimal L-asparaginase-based regimen for HIV-positive patients is not established. Options include SMILE, GLIDE, and P-GemOxD. The SMILE regimen carries significant myelosuppression and hepatotoxicity. The GLIDE regimen, developed at our institution, achieved a 78.3% CR rate but requires an 11-day infusion, prolonging hospitalization and increasing infectious risk. Through multidisciplinary deliberation, we selected P-GemOxD based on: (1) its 5-day course minimizes inpatient exposure; (2) a multicenter study confirmed a 73% 3-year OS with manageable toxicity; and (3) its shorter duration and lower hepatic impact theoretically reduce interactions with ART. The patient received full-dose P-GemOxD despite a CD4^+^T cell nadir of 198 cells/μL—below the 200/μL threshold that often prompts dose reduction. Evidence suggests that full-dose curative regimens are appropriate when CD4^+^T cells count exceed 50–100 cells/μL. The patient had been receiving SMZ-TMP prophylaxis since HIV diagnosis, which was continued throughout chemotherapy, and no pneumocystis occurred. The MDT weighed this evidence against the patient’s individual risk profile and prioritized lymphoma control, a decision validated by the patient’s excellent tolerance and eventual CR (7, 8, 12–14). Importantly, at 9 months after radiotherapy completion, the patient’s CD4^+^T cell counts had risen to 255 cells/μL with sustained HIV-1 RNA suppression.

Limitations include limited generalizability, selection bias from the patient’s financial-driven ART choice, and short follow-up. Nonetheless, data on ART-chemotherapy management in HIV-associated ENKTCL-NT remain scarce, and our case is among the few to document the use of P-GemOxD specifically with an LPV/r-based ART, providing a practical management framework when INSTI-based regimens are not accessible. More broadly, this case illustrates that the complexity of HIV-associated malignancies, where antiviral and antineoplastic therapies intersect, demands a structured, team-based approach. In our experience, the MDT model facilitated the real-time reconciliation of competing priorities, from regimen selection to dose intensity to drug interaction surveillance. Although a single case cannot establish a casual relationship between dual remission in oncology and virology and specific treatment decisions, our observations suggest that this multidisciplinary framework may offer an effective strategy for addressing similar clinical challenges.

## Data Availability

The original contributions presented in the study are included in the article/**Supplementary Material**. Further inquiries can be directed to the corresponding authors.
