# Supplementary material for: HIV-associated extranodal NK/T cell lymphoma, nasal type: a case report and literature review
Source: Front Immunol. 2026 Oct 7;17:1954471. doi: 10.3389/fimmu.2026.1954471 (PMC13683803; doi:10.3389/fimmu.2026.1954471)
Supplement: Supplementary file 1 [file Supplementaryfile1.pdf]

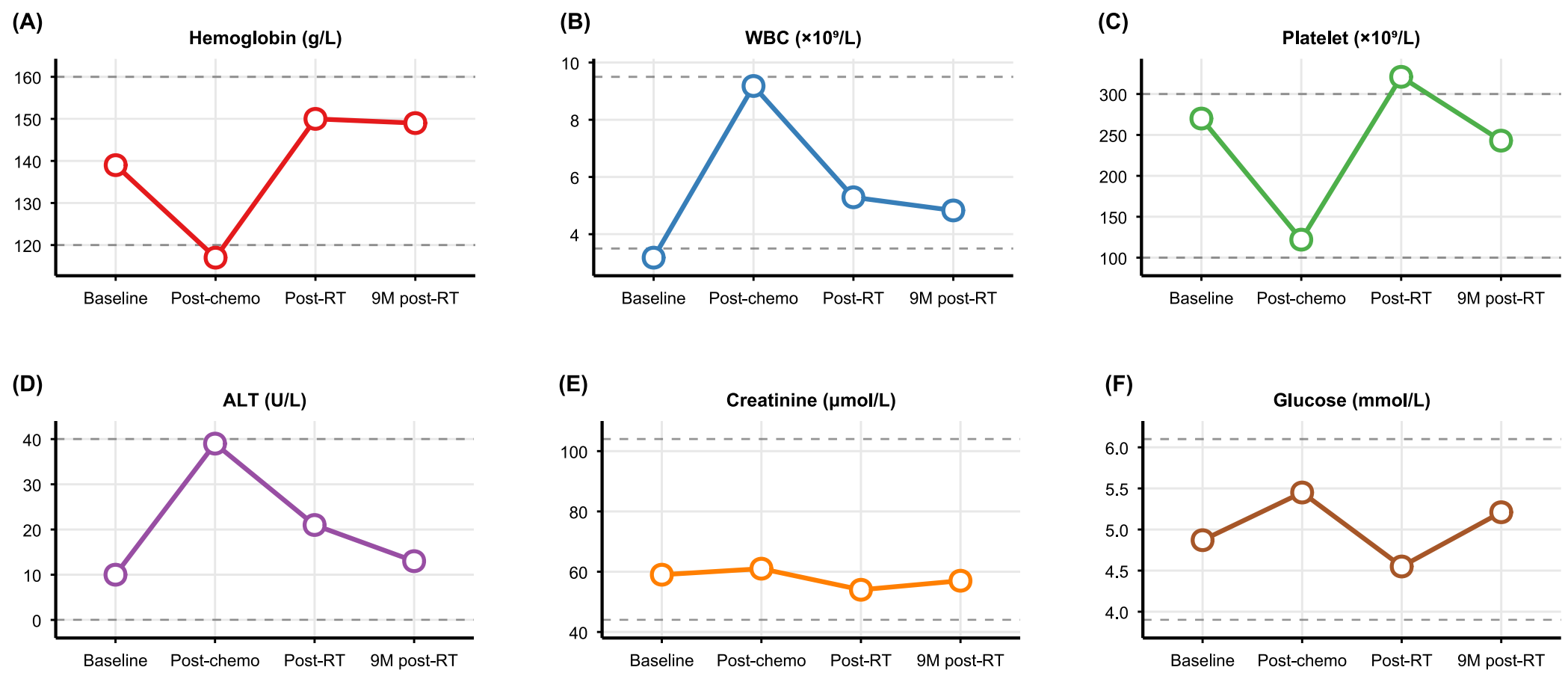

Figure S1. Longitudinal changes in laboratory parameters before and after chemoradiotherapy.

(A) Hemoglobin (HB);

(B) White blood cell count (WBC);

(C) Platelet count (PLT);

(D) Alanine aminotransferase (ALT);

(E) Creatinine (CREA);

(F) Fasting glucose (GLU).

Data are presented as measured values at four time points: Baseline (admission), Post-chemo (after chemotherapy completion), Post-RT (after radiotherapy completion), and 9M post-RT (9 months after radiotherapy completion). Dashed horizontal lines indicate the normal reference ranges for each parameter: HB 120–160 g/L, WBC 3.5–9.5 × 10^9/L, PLT 100–300 × 10^9/L, ALT 0–40 U/L, CREA 44–104 μmol/L, GLU 3.9–6.1 mmol/L. Points represent individual measurements, and lines connect sequential time points to illustrate temporal trends. HB, WBC, and PLT were decreased after chemotherapy but recovered following radiotherapy. ALT transiently elevated after chemotherapy and normalized thereafter. CREA and GLU remained within normal ranges throughout the treatment course.
